# Comparing the selective and co-selective effects of different antimicrobials in bacterial communities

**DOI:** 10.1016/j.ijantimicag.2019.03.001

**Published:** 2019-06

**Authors:** Aimee K. Murray, Lihong Zhang, Jason Snape, William H. Gaze

**Affiliations:** a European Centre for Environment and Human Health, University of Exeter Medical School, Environment & Sustainability Institute, Penryn Campus, Penryn, Cornwall, TR10 9FE; b AstraZeneca Global Environment, Alderly Park, Macclesfield

**Keywords:** Antibiotic, Antimicrobial, Biocide, Resistance, Evolution, Metagenomics

## Abstract

Bacterial communities are exposed to a cocktail of antimicrobial agents, including antibiotics, heavy metals and biocidal antimicrobials such as quaternary ammonium compounds (QACs). The extent to which these compounds may select or co-select for antimicrobial resistance (AMR) is not fully understood. In this study, human-associated, wastewater-derived bacterial communities were exposed to either benzalkonium chloride (BAC), ciprofloxacin or trimethoprim at sub-point-of-use concentrations for one week to determine selective and co-selective potential. Metagenome analyses were performed to determine effects on bacterial community structure and prevalence of antibiotic resistance genes (ARGs) and metal or biocide resistance genes (MBRGS). Ciprofloxacin had the greatest co-selective potential, significantly enriching for resistance mechanisms to multiple antibiotic classes. Conversely, BAC exposure significantly reduced relative abundance of ARGs and MBRGS, including the well characterised *qac* efflux genes. However, BAC exposure significantly impacted bacterial community structure. Therefore BAC, and potentially other QACs, did not play as significant a role in co-selection for AMR as antibiotics such as ciprofloxacin at sub-point-of-use concentrations in this study. This approach can be used to identify priority compounds for further study, to better understand evolution of AMR in bacterial communities exposed to sub-point-of-use concentrations of antimicrobials.

## Highlights

• Selective and co-selective effects of antimicrobials were compared for the first time.
• Ciprofloxacin and trimethoprim were more co-selective than selective.
• Benzalkonium chloride (BAC) did not select for antibiotic/metal/*qac* resistance genes.
• Metagenomics could identify highly co-selective compounds for further study.

## 1 Introduction

Antimicrobial resistance (AMR) occurs naturally in a variety of environments [1] but anthropogenic use, overuse and misuse of antibiotics and other antimicrobials has selected for increased levels of resistance [2]. Direct selection for AMR can arise when bacteria are exposed to a single compound; for example, exposure to ciprofloxacin can result in increased numbers of bacteria harbouring a *gyrA* mutation that confers resistance to ciprofloxacin [3]. Conversely, co-selection is indirect selection for a resistant phenotype that can occur via two mechanisms: cross-resistance or co-resistance [4]. Cross-resistance occurs when one resistance gene can confer resistance to many antimicrobials [4]. For example, the *qac* resistance genes encode multidrug efflux pumps that efflux many different quaternary ammonium compounds (QACs) [5]. Therefore, exposure to one of these compounds would result in selection for the efflux gene. Co-resistance is when a resistance gene will be maintained/selected if it is genetically linked to another gene (though not necessarily a resistance gene) that is under positive selection [4]. *Qac* genes may also be co-selected via co-resistance as they are often located on integrons, which in turn can carry a vast range of antibiotic resistance genes (ARGs) [6], [7].

There are two main types of antimicrobial agents. Antibiotics are used therapeutically and prophylactically in humans and animals, and as growth promoters in animal husbandry in some parts of the world. Antibiotics are not fully metabolised by humans and animals, and in some cases >90% of an antibiotic can be excreted in an active form [8]. Other compounds with antimicrobial effects include biocides, such as QACs, and heavy metals. QACs are used widely for equipment sterilisation, product preservation and surface decontamination in a variety of settings, including in hospitals, farms and households [9]. Heavy metals are required by most bacteria for growth but are toxic at high concentrations. Heavy metals are used in animal feed [10], and in antibacterial products, such as wound dressings [11]. They can accumulate in the environment due to industrial contamination [12]. In theory, each antimicrobial has the potential to co-select for resistance to another.

Antibiotic concentration gradients exist within human, animal and environmental microbiomes from point-of-use until they are diluted to extinction. Several studies have indicated that sub-inhibitory concentrations of antibiotics exhibit biological effects and can even select for AMR [3], [13], [14], [15], but few studies have looked at co-selective effects. The selective and co-selective effects of different antimicrobials at sub-point-of-use concentrations have not previously been compared within bacterial communities. In this study, we exposed a wastewater-derived bacterial community (including gut microbiome bacteria and the World Health Organization [WHO] critically important Enterobacteriaceae [16]*,* from many individuals) to the QAC biocide benzalkonium chloride (BAC), ciprofloxacin or trimethoprim at sub-point-of-use concentrations in serial passage experiments for 7 days. BAC was chosen as it would likely co-select for resistance via cross-resistance and co-resistance via the *qac* multidrug efflux genes. Ciprofloxacin was included in this study as it has been shown to be selective at sub-inhibitory concentrations [3]. Finally, trimethoprim was chosen as *dhfr* genes are one of the most common antibiotic resistance genes associated with class 1 integrons [6], and may co-select for integron-borne resistance via co-resistance.

Metagenome analyses of communities exposed to these antimicrobials were performed to determine effects on bacterial community structure and prevalence of ARGs and metal or biocide resistance genes (MBRGs). Our findings indicate BAC, unlike ciprofloxacin, was not a potent co-selective compound in this experimental system. We identified potentially important gene targets for tracking QAC resistance, in addition to the most well-studied *qac* efflux genes. Finally, results illustrated the potential for metagenome analyses to identify priority antimicrobial compounds for further study, based on their selective and co-selective potential and corresponding threat to human health.

## 2 Materials and Methods

### 2.1 Evolution experiment

Untreated wastewater was collected from a sewage treatment plant (population equivalent of 43 000) in October 2015 and frozen at 50% v/v in 40% glycerol at -80°C until use. Frozen samples underwent two steps of centrifugation (3500 *x g* for 10 min) and resuspension in equal volume 0.85% sterile saline to minimise chemical and nutrient carry over.

There were three replicate microcosms for each antimicrobial. Compounds used were BAC (8 mg/L), ciprofloxacin (0.5 mg/L) and trimethoprim (2 mg/L) at half the clinical breakpoint concentrations [17] for Enterobacteriaceae (ciprofloxacin and trimethoprim) or half the minimum inhibitory concentration (MIC) of the susceptible K12 *Escherichia coli* strain (BAC), as determined by the standard MIC plating method [18]. This was based on the assumption that a significant portion of the human-derived wastewater would include this family of bacteria, and based on a previous study in the same experimental model system where *E. coli* was the prominent detected species [19]. Antimicrobial-amended microcosms (n = 3 per antimicrobial, with n = 3 antimicrobial-free control) comprising 5 mL Iso-sensitest broth (Oxoid) and 1% v/v processed wastewater sample were incubated overnight at 37°C, shaking at 180 rpm.

Each day, 1% v/v of cultures were inoculated into fresh, antimicrobial-amended media. This was repeated for a total of 6 days. On the 7^th^ day, 1 mL culture was centrifuged (21 000 *x g*) for 2 min, resuspended in equal volume of 20% glycerol, and stored at -80°C.

### 2.2 DNA extraction, clean up and sequencing

Total bacterial DNA was extracted using the MoBio ultraclean kit, according to instructions but with the initial spin extended to 3 min. All DNA was stored at -20°C until use.

DNA was cleaned and concentrated using Ampure™ beads, as previously described [20]. Nextera XT libraries were prepared and sequenced on the Illumina HiSeq 2500 platform by Exeter Sequencing Service (ESS), generating 300 bp paired end reads.

### 2.3 Metagenome analyses

Successful removal of adaptor sequences and low-quality reads was performed with Skewer [21] and confirmed with MultiQC [22] before and after trimming. The number of reads for each sample after trimming are reported in the Supplementary Data (Table S1).

Extraction and analyses of 16S rRNA sequences were performed as described previously [20]. Briefly, reads were paired with FLASH version 2 [23] and 16S rRNA reads were extracted with MetaPhlan2 [24]. Community diversity visualisation was performed with HClust2 [25] using Bray Curtis distance measurements between samples and features (species). Biomarker species/genera were identified with LEfSe (linear discriminant analysis effect size) [26].

ARGs were identified with the ARGs-OAP pipeline, which identifies ARGs at the antibiotic class and within class level, and normalises these hits to both the length of the ARG itself and either parts per million, 16S rRNA copy number or cell number to derive ARG relative abundance [27]. The default cut-off values for ARG assignment were used (25 amino acid, e-value of 1e-07 and 80% identity). MBRGs were identified through BacMet Scan against the experimentally confirmed BacMet database, using default search parameters and cut-off values [28]. All ARGs and MBRGs hits were normalised to hits per million reads. Heatmaps were generated using various python packages [29], [30], [31].

### 2.4 Statistical methods

Normally distributed data were analysed with parametric one-way ANOVA and Tukey post-hoc tests. Non-normally distributed data that could not be transformed with log or square root functions into a normal distribution underwent non-parametric Kruskal Wallis and Dunn's tests. *P-*values for the post-hoc Tukey test or Dunn's test are reported. Spearman's rank correlation between hits of ARGs and MBRGs per million reads determined whether there was a positive or negative correlation for all three tested antimicrobials.

## 3 Results

### 3.1 Effects on community structure

**Fig. 1:**
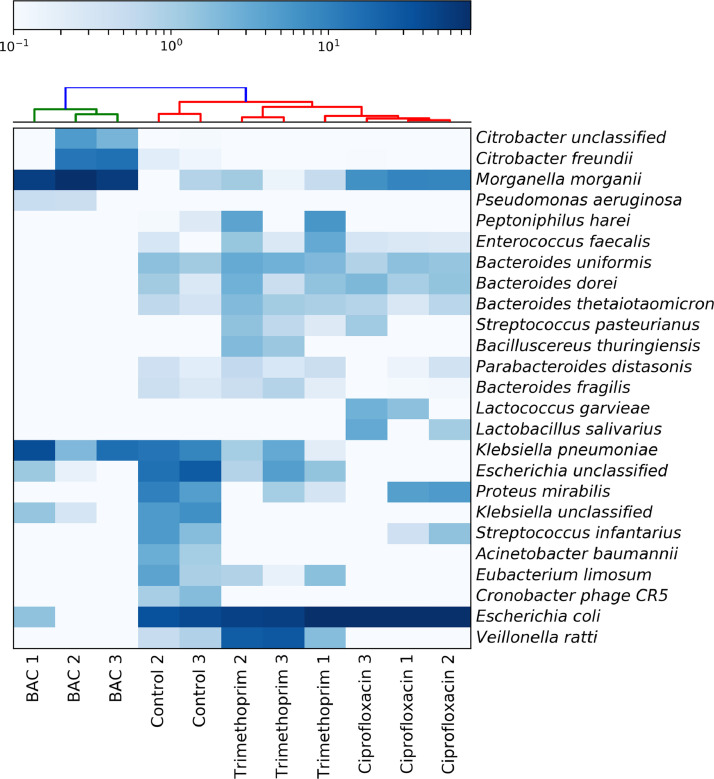
Heatmap showing the 25 species with highest relative abundance for each biological replicate within each antimicrobial treatment, as determined with MetaPhlan2, using Bray-Curtis distance measurements for samples and features (species).

A wastewater bacterial community was exposed to sub-point-of-use concentrations of either BAC (8 mg/L), ciprofloxacin (0.5 mg/L) or trimethoprim (2 mg/L) equating to half the BAC MIC for susceptible *E. coli* and half the clinical breakpoint for the two antibiotics [17]. Metagenome analyses were performed on biological replicates for each of the antimicrobial treatments and from the cultured control. The top 25 detected species for each replicate are shown in Fig. 1 (for all detected species, see Figure S1).

There were a total of 26–62 bacterial species detected in metagenomes across treatments (Fig. 1 and Figure S1). These included mostly facultative anaerobes as well as some microaerophilic bacteria. Linear Discriminant Analysis Effect Size (‘LEfSe’) identifies ‘features’ (such as bacterial species or genera) that can be used to highlight differences between, for example, experimental treatments, different body sites or environments by combining statistical significance testing with tests that consider biological consistency and effect relevance [26]. LEfSe was used to identify species significantly associated with different treatments (species ‘biomarkers’). LEfSe defined the control treatment as having the greatest number of biomarker bacterial genera, with *Streptococcus, Staphylococcus, Acinetobacter, Eggerthella, Enterobacter* and *Cronobacter* species all significantly associated with the control treatment, indicating equal representation of Gram-negative and Gram-positive biomarker genera (Table S2).

BAC had the greatest effect on community structure, resulting in complete loss of 18 species relative to the control (Fig. 1). Only 5 of the original 28 bacterial genera persisted in BAC treatments, these were Gram-negative genera, including *Citrobacter, Escherichia, Klebsiella, Morganella* and *Pseudomonas. P. aeruginosa, K. pneumoniae* and *M. morganii* were determined as biomarkers in the BAC treatment (Figure S1, Table S2). Interestingly, the opportunistic pathogen *P. aeruginosa* was below the limit of detection in the control treatment, but was enriched to a high abundance in the BAC treatment, thus indicating strong selection for this often intrinsically resistant organism. Only two bacterial genera were biomarkers for the ciprofloxacin treatment: *Escherichia* and *Lactobacillus* (Table S2). Trimethoprim had three biomarker genera, including *Veillonella, Bacteriodes* and *Bifidobacterium* (Table S2). Therefore, unlike in the BAC treatment, some Gram-positive bacteria persisted following ciprofloxacin and trimethoprim exposure.

Generally, *E. coli* was the most abundant species in control and antibiotic treatments, though ciprofloxacin and trimethoprim exposure resulted in slight decreases in *E. coli* abundance compared with the control. In the BAC treatment, *E. coli* relative abundance was much lower and was only detected in a single treatment replicate (Fig. 1).

### 3.2 Co-selective potential of different antimicrobials for ARGs

**Fig. 2:**
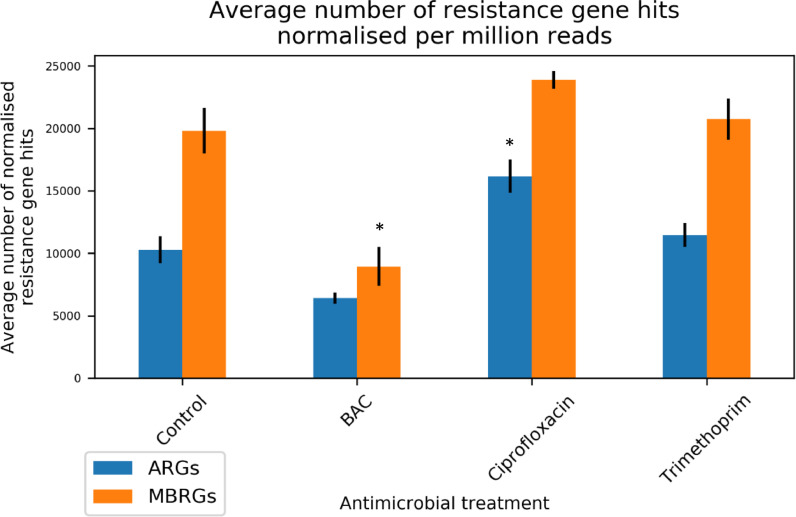
Total number of ARG/MBRG hits normalised per million reads (detected with ARGs-OAP and BacMetScan, respectively), averaged within treatment (n = 3 for antimicrobial treatments, n = 2 for the control). * = significant difference in numbers of hits relative to the control (*P* <0.05).

The ARGs-OAP pipeline [27] was used to identify ARGs within all treatment replicates. ARG hits were normalised to number of hits per million reads and summed per antimicrobial treatment. The total number of ARG hits was highest following ciprofloxacin exposure, with replicate number 3 having >18 000 ARG hits per million reads (Table S3). Overall, total number of ARG hits per million reads was significantly different to the control (*P* = 0.02, Fig. 2). However, the sum of ARGs in both BAC and trimethoprim treatments was not significantly different to the control.

**Fig. 3:**
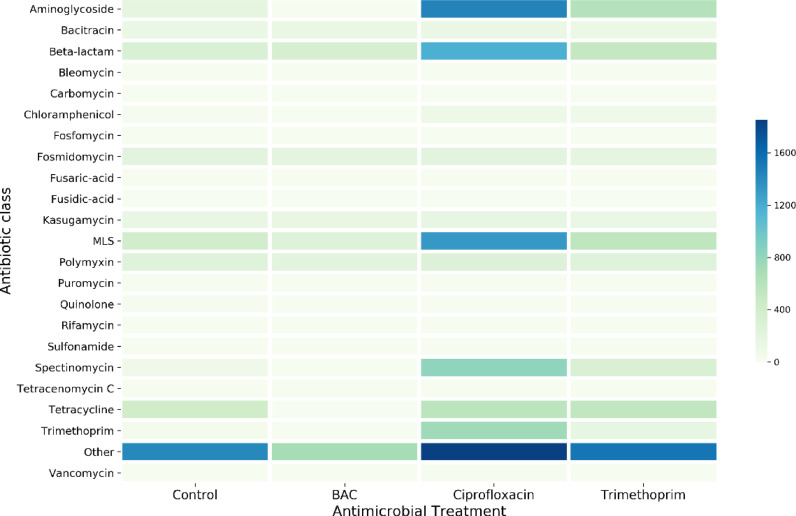
Heatmap showing average relative abundance of ARG hits (antimicrobial treatments n = 3, control n = 2) detected for different antibiotic classes with the ARGs-OAP pipeline. Numbers of hits are normalised per million reads. ‘MLS’ = Macrolide-Lincosamide-Streptogramin resistance. Multidrug resistance hits are excluded due to extremely high abundance (Figure S2).

Multidrug resistance mechanisms were the most abundant type of resistance mechanism in all treatments (Figure S2), but there was little variability between treatments. Ciprofloxacin was the most co-selective antimicrobial of the three tested, as there was significant enrichment for aminoglycoside (*P* = 0.011), beta-lactam (*P* = 0.016), chloramphenicol (*P* = 0.019), macrolide-lincosamide-streptogramin (‘MLS’, *P* = 0.035), sulphonamide (*P* = 0.033), trimethoprim (*P* = 0.035) and vancomycin (*P* = 0.023) resistance genes compared with the control (Fig. 3). Conversely, no significant increases in any ARGs were observed following BAC treatment. Rather, BAC treatment resulted in significant decreases in multidrug resistance genes (*P* = 0.029) and genes conferring ‘unclassified’ resistance to other antibiotics (*P* = 0.008). Trimethoprim had little effect on relative abundance of ARGs, with the only significant increase observed for chloramphenicol resistance genes (*P* = 0.046) (Fig. 3).

Surprisingly, ARGs conferring resistance to ciprofloxacin or trimethoprim were not significantly enriched following exposure to either compound (e.g. significant enrichment of quinolone ARGs was not observed following ciprofloxacin exposure).

### 3.3 Co-selective potential of different antimicrobials for MBRGs

Finally, all metagenomes were screened against the experimentally confirmed BacMet database [28], which contains MBRGs. Again, total numbers of MBRG hits were normalised per million reads. There was a statistically significant positive relationship between total numbers of ARG and MBRG hits (*r* = 0.91, df = 9, *P* < 0.0001). The only significant difference for the sum of MBRGs after antimicrobial treatment compared with the control was for BAC, where total numbers of MBRGs decreased significantly compared with the control (*P* = 0.007, Fig. 2).

This was an interesting finding, combined with the lack of selection for ARGs. There are only three *qac* genes in the ARGs-OAP database [27], so it was expected that there would be a greater number of hits for *qac* genes and other QAC resistance mechanisms when searched against the BacMet database [28]. Currently in the BacMet database, there are 64 experimentally-confirmed BAC resistance mechanisms, 13 of which have been found on plasmids. Plasmid-encoded genes include *oqxA, oqxB,* all *qac* genes, and *sugE* (BacMet search, 7^th^ March 2018). The total number of *qac* genes*, oqxA/B* or *sugE* genes was compared between treatments and ciprofloxacin was found to significantly enrich *qac* genes (*P* = 0.034). Total hits for *oqxA/B* genes were significantly lower following ciprofloxacin exposure compared with the control (*P* = 0.016). The only plasmid-borne BAC resistance genes that increased in relative abundance following BAC exposure were the *oqxA/B* genes, although this increase was not significant. Surprisingly, *qac* genes and *sugE* genes decreased in relative abundance following treatment with BAC; again, these differences were not significant. There were no significant differences in total number of hits for *qac, sugE* or *oqx* genes between the control and trimethoprim treatment.

Detected chromosomally-encoded BAC resistance genes and their total number of hits were also investigated (Table 1). Only 6 of a possible 51 chromosomally-encoded BAC resistance genes were detected in any treatment in this study. The *acrE/envC* and *acrF/envD* efflux systems were common across all treatments and formed the largest portion of chromosomal BAC resistance gene hits. Also detected were the *abeS* and *adeT1* genes, which encode efflux pumps: these were found in the control treatment only and a single replicate of the BAC treatment, respectively. The *cpx* genes, *cpxA* and *cpxR*, were found in 2 BAC replicates and 1 trimethoprim replicate. When examining total hits for detected chromosomally-encoded BAC resistance genes, there was a significant decrease in hits in the BAC treatment compared with the control (*P* = 0.033).

**Table 1:** Total number of experimentally confirmed, chromosomally encoded BAC resistance gene hits detected in this study with BacMetScan, normalised against per million reads.

	*Control*	*BAC*	*Ciprofloxacin*	*Trimethoprim*
*Number of normalised hits*	500	225⁎⁎	736*	745*
	*Detected genes*			
*acrE/envC*	127	15	195	178
*acrF/envD*	371	90	541	482
*cpxA*	ND	63	ND	148
*cpxR*	ND	88	ND	107
*adeT1*	ND	1^1^	ND	ND
*abeS*	2	ND	ND	ND

Hits are average within antimicrobial treatments (n = 3 for antimicrobials, n = 2 for control).

⁎ significantly greater number of hits;

⁎⁎ significantly reduced number of hits, relative to the control. ND = Not detected.

1 detected in biological replicate 1 only.

## 4 Discussion

Bacterial communities are exposed to a variety of antimicrobial compounds. Previous observational studies have found correlative evidence to indicate QACs co-select for antimicrobial resistance in QAC polluted environments [32], [33]. More recent experimental studies have observed direct selection for QAC resistance in bioreactors of bacterial communities exposed to BAC [34], but did not investigate AMR co-selection. Currently, there are no studies that have examined the potential for biocides, such as QACs, to co-select for antibiotic resistance in bacterial communities and compared this to direct selection by antibiotic exposure.

Our findings that BAC exposure has significant effects on bacterial community structure, resulting in competitive exclusion of susceptible bacteria and clonal expansion of a few resistant bacterial species, agree with previous results [34]. At the end of this study, all bacteria in BAC treatment replicates were Gram-negative and comprised only 8 detected species. *E. coli* were almost fully outcompeted and were detected in only a single replicate, even though the exposure concentration was half the MIC for a susceptible *E. coli* laboratory strain. Although the starting inoculum metagenome was not sequenced in this study, the no antimicrobial control treatments control for potential effects on the community. Further studies should increase the sequencing frequency, so such dynamics can be better understood.

Previous work indicates QACs have a high predicted co-selective potential compared with other biocides and heavy metals, because of close genetic proximity of additional resistance mechanisms that could be co-selected by co-resistance [35]. Our experimental approach shows for the first time that the majority of ARGs and MBRGs are lost following BAC exposure at half the MIC for susceptible *E. coli* (relative to the control). There are two likely explanations for loss of ARGs/MBRGs that are not mutually exclusive. The first is that the resistance gene sequences enriched by BAC treatment are not currently deposited in the ARGs-OAP and BacMet databases. The second is that BAC at 8 mg/L enriches for intrinsically resistant organisms, which outcompete susceptible organisms, including those harbouring mobile resistance mechanisms (which may have increased fitness costs). The latter of these scenarios is supported by the enrichment of solely Gram-negative bacteria, which generally have elevated levels of resistance to QACs compared with Gram-positive bacteria, and by the analysis of mobile QAC resistance mechanisms (i.e. plasmid borne genes). There were no significant differences in the relative abundance of mobile QAC resistance genes between BAC and control treatments, including the well-characterised *qac* resistance genes. This finding indicates the need for continued efforts to identify potentially novel resistance genes that confer QAC resistance, because *qac* genes may not be as significant in QAC resistance as the literature indicates. This finding also indicates the potential utility of bacterial community analyses combined with ARGs/MBRG mining in determining the selective and co-selective potential of different antimicrobials. Intrinsically resistant organisms pose a considerably reduced risk to human health compared with bacteria that can readily transfer resistance, because their resistance mechanisms are not readily mobilisable. Therefore, a metagenome approach can be used to prioritise antimicrobials in terms of their potential risk to human health through identifying compounds with strong selective potential, compounds that readily co-select for many types of different resistance mechanisms, and whether these resistance mechanisms are likely to be harboured by intrinsically resistant organisms (indicated by lack of community diversity).

While BAC exposure resulted in loss of ARGs and MBRGs, ciprofloxacin treatment enriched relative abundance of ARGs to 7 different antibiotic classes. Resistance genes detected in this study are not necessarily all being expressed; however, this is not relevant for co-resistance (i.e. co-location) of genes on the chromosome or on mobile genetic elements. Our results combined with findings from other studies (that have shown selection can occur at very low concentrations of ciprofloxacin [3]) demonstrate the high selective and co-selective potential of ciprofloxacin and indicate further research on this antibiotic is required.

Trimethoprim exposure resulted in significant enrichment of only chloramphenicol resistance genes. Interestingly, trimethoprim-resistant species, such as *Pseudomonas aeruginosa,* were not selected for, which indicates their relative low fitness in this community compared with other resistant bacteria. Neither of the antibiotics directly selected for their own previously described resistance mechanisms (i.e., ciprofloxacin exposure did not result in significant increases in relative abundance of quinolone ARGs, nor trimethoprim in relative abundance of trimethoprim ARGs). This indicates a high abundance of genes conferring cross-resistance to more than one compound; presence of intrinsically resistant organisms (possibly outcompeting organisms with known ARGs); and/or an incomplete database. Functional studies aiming to identify novel resistance genes and their complete antimicrobial susceptibility profiles are still critical for improving our understanding of selection and co-selection. New techniques, such as emulsion, paired isolation and concatenation (EPIC) PCR [36], could be used to discern if resistance genes that are being selected for are present in only a few species (indicating selection of that species), or if they are widespread throughout the bacterial population (indicating potential for horizontal gene transfer).

## 5 Conclusions

In summary, selective and co-selective effects of different antimicrobials at sub-point-of-use concentrations were compared in this study for the first time. BAC exerted a relatively low selective pressure for AMR development in bacterial communities, relative to antibiotics, although this may be in part due to a high diversity of uncharacterised resistance genes, which were undetected. Ciprofloxacin was shown to be the most co-selective compound tested, and should be prioritised for further study to investigate the risk for selection and co-selection in a variety of settings. A metagenome approach to quantify the risk of selection for AMR can be useful to identify additional priority compounds based on their selective and co-selective potential, and whether this resistance is likely to be readily mobilisable.

## Appendix Supplementary materials

Image, application 1
