# Supplementary material for: Comparing the selective and co-selective effects of different antimicrobials in bacterial communities
Source: Int J Antimicrob Agents. 2019 Jun;53(6):767–73. doi: 10.1016/j.ijantimicag.2019.03.001 (PMC6546120; doi:10.1016/j.ijantimicag.2019.03.001)
Supplement: Supplementary file 1 [file mmc1.docx]

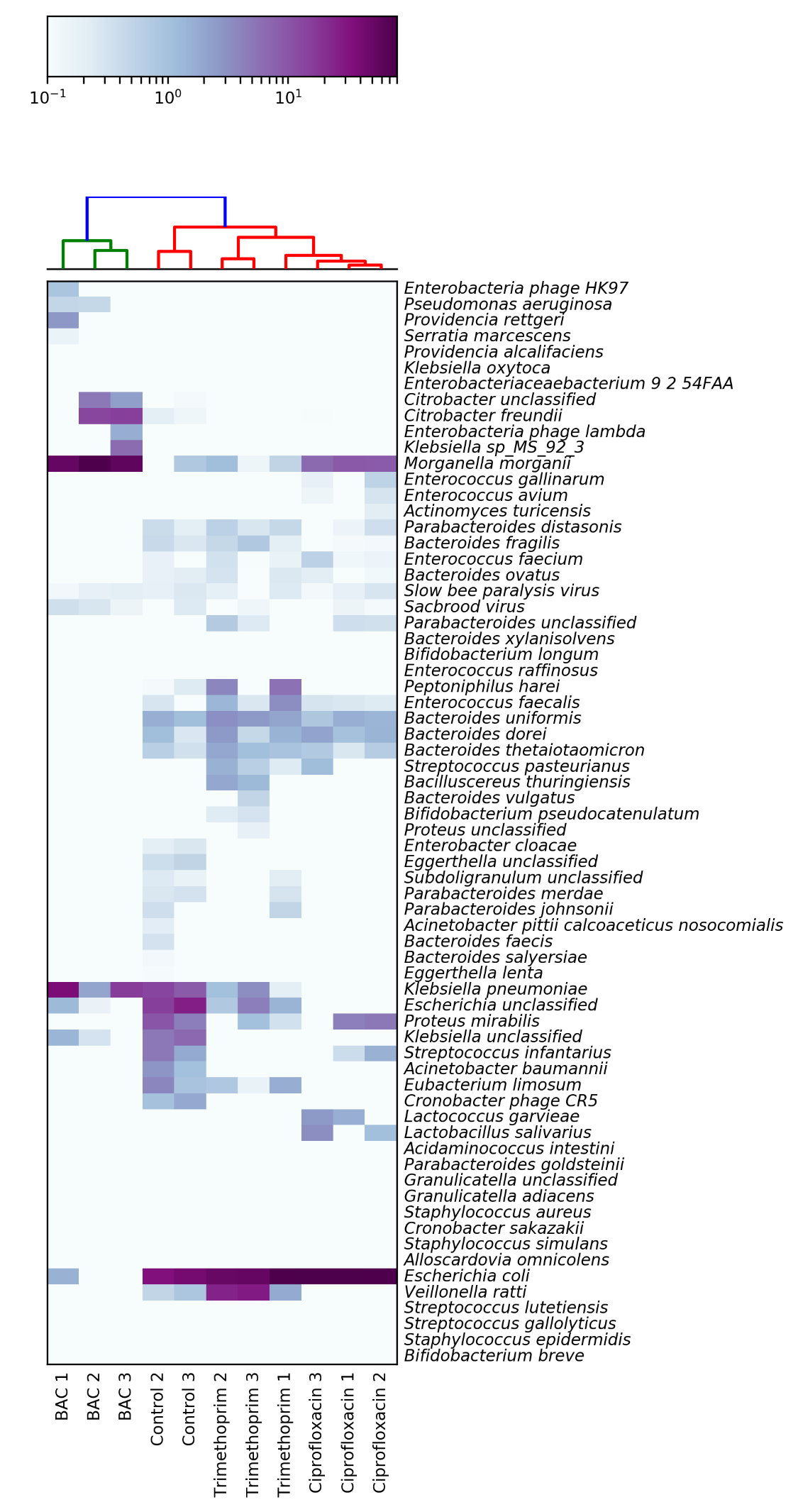


Figure S1. Clustermap showing all bacterial species detected in this study across all treatments, as determined with MetaPhlan2, using Bray-Curtis distance measurements for samples and features (species) and clustering for samples only.


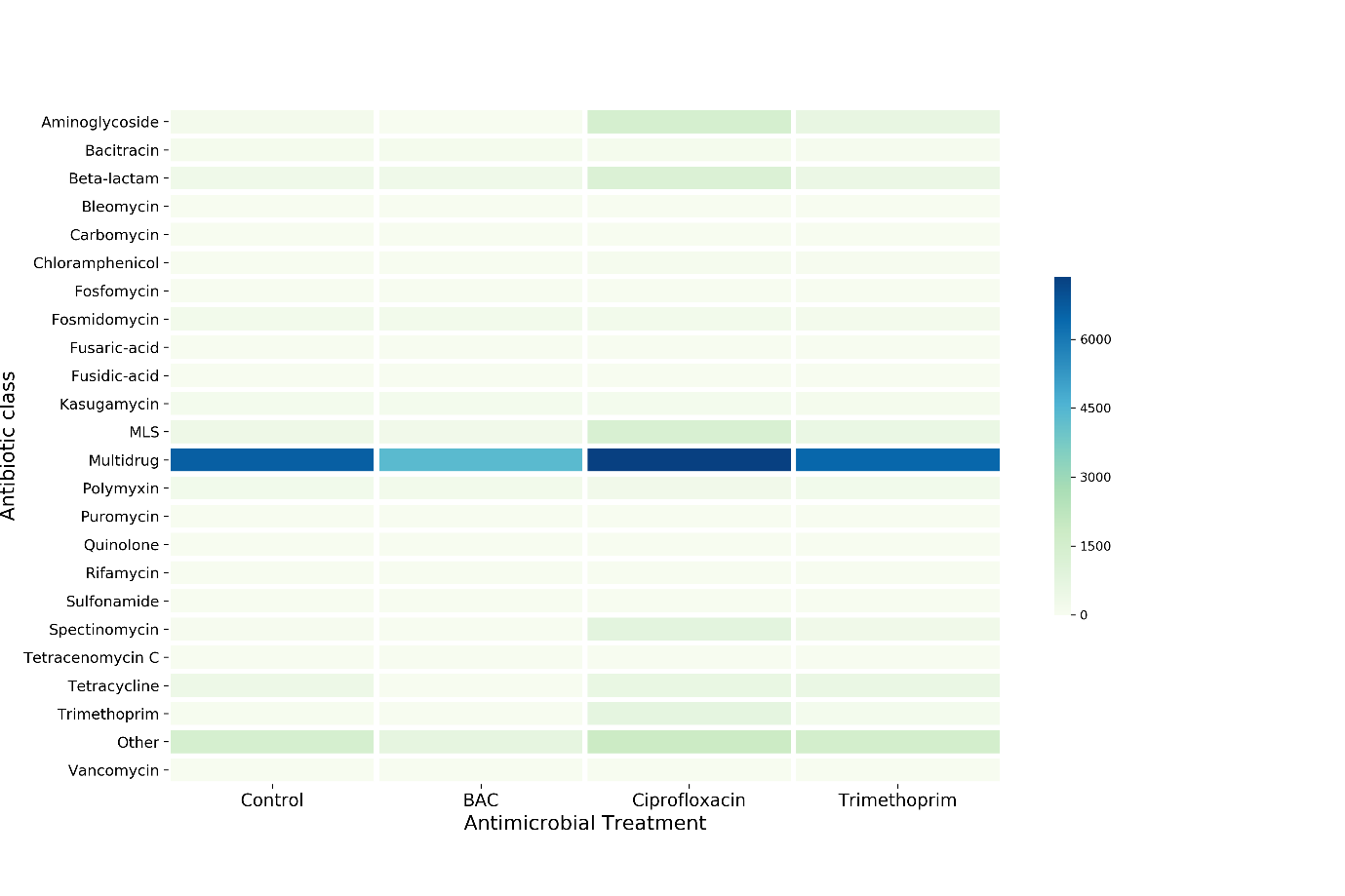


Figure S2. Heatmap showing average relative abundance of all ARG hits (antimicrobial treatments n=3, control n=2) conferring resistance to different antibiotic classes (including multidrug resistance genes) with the ARGs-OAP pipeline. Numbers of hits are normalised per million reads. ‘MLS’ = Macrolide-Lincosamide-Streptogramin resistance.

Table S1. Number of reads per replicate, per sample, after quality trimming and removal of adaptor sequences. Number of reads in mega base pairs (Mbp) rounded to 2 decimal places.

| Antimicrobial treatment | Replicate number | No. reads (Mbp) | Average no. reads (Mbp) per treatment |
| --- | --- | --- | --- |
| Benzalkonium Chloride | 1 | 3.30 |  |
| Benzalkonium Chloride | 2 | 1.78 |  |
| Benzalkonium Chloride | 3 | 5.83 | 3.64 |
| Ciprofloxacin | 1 | 2.52 |  |
| Ciprofloxacin | 2 | 2.39 |  |
| Ciprofloxacin | 3 | 3.55 | 2.82 |
| Trimethoprim | 1 | 1.76 |  |
| Trimethoprim | 2 | 1.63 |  |
| Trimethoprim | 3 | 2.55 | 1.98 |
| Control | 2 | 5.20 |  |
| Control | 3 | 4.01 | 4.61 |

Table S2. Linear Discriminant Analysis Effect Size (LEfSe) results, showing features significantly associated with each antimicrobial treatment, in order from highest LDA score (log 10) to lowest within treatment. Only fully classified features are reported. Classification key: ‘p’ = Phylum, ‘c’ = Class, ‘o’ = Order, ‘f’ = Family, ‘g’ = Genera, ‘s’ = Species.

| **Treatment** | **Feature** | **Classification** | **LDA score (log 10)** |
| --- | --- | --- | --- |
| **Trimethoprim** | Firmicutes | p | 5 |
|  | Veillonellaceae | f | 4 |
|  | Negativicutes | c | 4 |
|  | Selemondales | o | 4 |
|  | *Veillonella* | g | 4 |
|  | *Veillonella ratii* | s | 4 |
|  | *Bacteriodetes* | g | 4 |
|  | Bacteriodia | c | 4 |
|  | Bacteriodales | o | 4 |
|  | Bacteriodaceae | f | 4 |
|  | *Bacteriodes uniformis* | s | 4 |
|  | *Bacteriodes thetaiotaomicron* | s | 3 |
|  | *Bifidobacterium pseudocatenulatum* | s | 3 |
|  | *Bacteriodes fragilis* | s | 3 |
|  | *Parabacteriodes distasonis* | s | 3 |
|  | *Bifidobacterium* | g | 3 |
|  | Bifidobacteriaceae | f | 3 |
|  | Bifidobacteriales | o | 3 |
|  | *Enterococcus raffinosus* | s | 2 |
| **Control** | *Escherchia unclassified* | s | 4 |
|  | *Streptococcus infantarius* | s | 4 |
|  | Streptococcaceae | f | 4 |
|  | *Streptococcus* | g | 4 |
|  | *Acinetobacter baumannii* | s | 4 |
|  | *Acinetobacter* | g | 4 |
|  | Pseudomondales | o | 4 |
|  | Moraxellaceae | f | 3 |
|  | *Cronobacter phage CR5* | s | 3 |
|  | *Bacteriodes faecis* | s | 3 |
|  | *Myoviridae* | g | 3 |
|  | *Acinetobacter pittii calcoaceticus nosocomialis* | s | 3 |
|  | *Eggerthella lenta* | s | 3 |
|  | Coriobacteriaceae | f | 3 |
|  | *Staphylococcus* | g | 3 |
|  | Coriobacteriales | o | 3 |
|  | *Enterobacter cloacae* | s | 3 |
|  | *Eggerthella* | g | 3 |
|  | *Cronobacter sakazakii* | s | 3 |
|  | *Enterobacter* | g | 3 |
|  | Actinobacteria | p | 3 |
|  | *Cronobacter* | g | 3 |
|  | Staphylococcaceae | f | 2 |
| **Ciprofloxacin** | *Escherchia* | g | 5 |
|  | *Escherchia coli* | s | 5 |
|  | Lactobacillaceae | f | 5 |
|  | *Lactobacillus salivarius* | s | 3 |
|  | *Lactobacillus* | g | 3 |
|  | *Enterococcus gallinarum* | s | 3 |
| **BAC** | Gammaproteobacteria | c | 5 |
|  | Enterobacteriales | o | 5 |
|  | *Morganella morganii* | s | 5 |
|  | Proteobacteria | p | 5 |
|  | *Morganella* | g | 5 |
|  | Enterobacteriaceae | f | 4 |
|  | *Klebsiella* | g | 4 |
|  | *Klebsiella pneumoniae* | s | 4 |
|  | *Pseudomonas* | g | 3 |
|  | Pseudomonadaceae | f | 3 |
|  | *Pseudomonas aeruginosa* | s | 3 |

Table S3. Number of ARG and MBRG hits identified with the ARGs-OAP pipeline and BacMetScan per replicate (‘Rep’) for the antimicrobial and control treatments, normalised per million reads.

|  | BAC | | | CIPROFLOXACIN | | | cONTROL | | tRIMETHOPRIM | | |
| --- | --- | --- | --- | --- | --- | --- | --- | --- | --- | --- | --- |
| rEP | 1 | 2 | 3 | 1 | 2 | 3 | 2 | 3 | 1 | 2 | 3 |
| Sum ARGS | 6850 | 5498 | 6859 | 14787 | 14900 | 18818 | 9175 | 11347 | 13280 | 10107 | 10968 |
| Sum mbrGS | 8510 | 6466 | 11829 | 23364 | 23007 | 25296 | 17996 | 21632 | 23996 | 18630 | 19546 |
